# Hexaaqua­cobalt(II) bis­(5-acetyl-2-hy­droxy­benzoate) dihydrate

**DOI:** 10.1107/S1600536811046678

**Published:** 2011-11-09

**Authors:** Li-Jun Han, Shu-Ping Yang, Lin-Lin Fu, Hai-Lei Gao

**Affiliations:** a Department of Mathematics and Science, Huaihai Institute of Technology, Lianyungang 222005, People’s Republic of China; b Department of Chemical Engineering, Huaihai Institute of Technology, Lianyungang 222005, People’s Republic of China

## Abstract

In the title compound, [Co(H_2_O)_6_](C_9_H_7_O_4_)_2_·2H_2_O, the Co^2+^ cation lies on a twofold rotation axis and is coordinated by six water mol­ecules in a distorted octa­hedral geometry. In the 5-acetyl-2-hy­droxy­benzoate anion, the hy­droxy group links with the carboxyl­ate group *via* an intra­molecular O—H⋯O hydrogen bond and the acetyl group is twisted to the benzene ring at a dihedral angle of 16.99 (12)°. In the crystal structure, the cations, anions and water mol­ecules are linked by extensive O—H⋯O hydrogen bonding.

## Related literature

For related cobalt salts, see: Wang *et al.* (2011 ▶); Zhang *et al.* (2011 ▶).
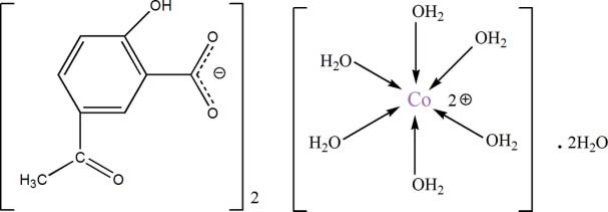


## Experimental

### 

#### Crystal data

[Co(H_2_O)_6_](C_9_H_7_O_4_)_2_·2H_2_O
*M* *_r_* = 561.35
Orthorhombic, 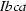

*a* = 10.6238 (10) Å
*b* = 13.6271 (12) Å
*c* = 33.318 (3) Å
*V* = 4823.5 (8) Å^3^
*Z* = 8
Mo *K*α radiation
μ = 0.79 mm^−1^
*T* = 298 K
0.40 × 0.30 × 0.30 mm

#### Data collection

Bruker APEXII CCD area-detector diffractometer
Absorption correction: multi-scan (*SADABS*; Bruker, 2008 ▶) *T*_min_ = 0.743, *T*_max_ = 0.798
19647 measured reflections
3036 independent reflections
2508 reflections with *I* > 2σ(*I*)
*R*_int_ = 0.025

#### Refinement

*R*[*F* ^2^ > 2σ(*F* ^2^)] = 0.038
*wR*(*F* ^2^) = 0.110
*S* = 1.03
3036 reflections
184 parameters
12 restraints
H atoms treated by a mixture of independent and constrained refinement
Δρ_max_ = 0.50 e Å^−3^
Δρ_min_ = −0.38 e Å^−3^

### 

Data collection: *APEX2* (Bruker, 2008 ▶); cell refinement: *SAINT* (Bruker, 2008 ▶); data reduction: *SAINT*; program(s) used to solve structure: *SHELXS97* (Sheldrick, 2008 ▶); program(s) used to refine structure: *SHELXL97* (Sheldrick, 2008 ▶); molecular graphics: *DIAMOND* (Brandenburg & Berndt, 1999 ▶); software used to prepare material for publication: *SHELXL97*.

## Supplementary Material

Crystal structure: contains datablock(s) I, global. DOI: 10.1107/S1600536811046678/xu5375sup1.cif

Structure factors: contains datablock(s) I. DOI: 10.1107/S1600536811046678/xu5375Isup2.hkl

Additional supplementary materials:  crystallographic information; 3D view; checkCIF report

## Figures and Tables

**Table 1:** Selected bond lengths (Å)

Co1—O1	2.0888 (15)
Co1—O2	2.0985 (16)
Co1—O3	2.0900 (15)

**Table 2:** Hydrogen-bond geometry (Å, °)

*D*—H⋯*A*	*D*—H	H⋯*A*	*D*⋯*A*	*D*—H⋯*A*
O1—H1*A*⋯O4^i^	0.84 (1)	1.93 (1)	2.766 (2)	179 (3)
O1—H1*B*⋯O8	0.83 (1)	1.91 (1)	2.728 (3)	168 (2)
O2—H2*A*⋯O4	0.84 (1)	1.83 (1)	2.667 (2)	172 (3)
O2—H2*B*⋯O8^ii^	0.83 (1)	2.25 (1)	3.069 (3)	171 (3)
O3—H3*A*⋯O5	0.84 (1)	1.85 (1)	2.680 (2)	176 (2)
O3—H3*B*⋯O6^iii^	0.83 (1)	1.93 (1)	2.752 (2)	172 (3)
O7—H7⋯O5	0.82	1.79	2.518 (2)	147
O8—H8*A*⋯O6^i^	0.83 (1)	2.17 (1)	2.996 (2)	171 (3)
O8—H8*B*⋯O1^iv^	0.84 (1)	2.59 (2)	3.267 (3)	139 (3)
O8—H8*B*⋯O3^iv^	0.84 (1)	2.37 (3)	3.054 (3)	139 (3)

Symmetry codes: (i) 
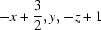
; (ii) 
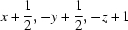
; (iii) 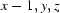; (iv) 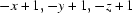.

**Fig. 1.**
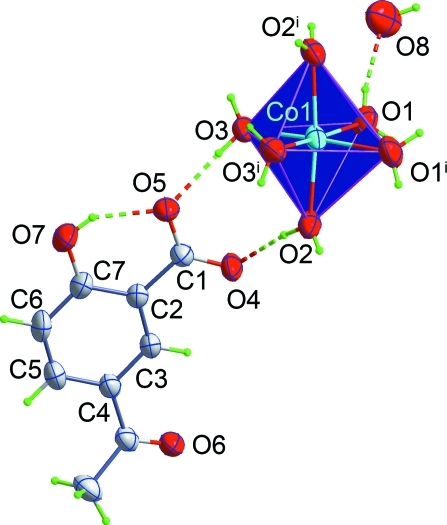
The asymmetric unit of the title structure and coordinated octahedron, showing the atom-numbering scheme. Displacement ellipsoids are drawn at the 50% probability level. Hydrogen bonds are shown as dashed lines. [Symmetric code:(i). -x + 1, -y + 1/2, z.].

## supplementary crystallographic information

### Comment

The crystal structures of hexaaquacobalt(II) carboxylate hydrate have been decribed (Zhang *et al.*, 2011; Wang *et al.*, 2011). We obtained unexpectedly the crystal structure of the title hexaaquacobalt(II) bis(5-acetyl-2-hydroxybenzoate) dihydrate in the preparation of cobalt(II) 5-acetyl-2-hydroxybenzoate complex, we report here the crystal structure of the title salt.

In the molecule, the asymmetric unit consist of half cobalt atom, three coordinated water molecules, one benzoic anion and one uncoordinated water molecule(Fig. 1), forming an axisymmetric structure and a coordinated octahedron of six water molecules around metal cobalt centrer generated by 2-fold rotation axis (1/2 1/4 *z*) across the Co1 atom, the midpoint of atoms O1 and O1^i^ and the midpoint of atoms O3 and O3^i^ [symmetry code: (i). 1 - *x*, 1/2 – *y*, *z*].

The equatorial plane of the octahedron is defined by atoms O1, O3, O1^i^ and O3^i^ with deviations of 0.0666 (12)Å for atom O1 and 0.0624 (11)Å for atom O3, and atom Co1 is located in the equatorial plane accurately; axis positions are occupied by atoms O2 and O2^i^. The equatorial Co — O bond distances are 2.0888 (15) and 2.0900 (15) Å, axial Co — O bond distance is 2.0985 (16) Å, axial O — Co — O bond angle O2 — Co1 — O2^i^ = 173.95 (7)°, the maximum equatorial O — Co — O bond angle O1 — Co1 — O3^i^ = 174.85 (6)°, the all other O — Co — O bond angles range from 87.42 (6) to 95.11 (6)°(Table 1).

In crystal structure, cations, anions and uncoordinated water molecules are linked into a two-dimensional crystal structure by O — H ··· O hydrogen bonds (Table. 2), neighbouring two-dimensional structure exist no any O—H···O hydrogen bond interactions.

### Experimental

Cobalt dichloride 0.47 g (2 mmol) was added to the solution (ethanol:water = 3:1, pH = 7) containing 5-acetyl-2-hydroxybenzoic acid 0.72 g (4 mmol) and sodium hydroxide 0.16 g (4 mmol). The reaction mixture was stirred for 2 h at 333–343 K and then the solution was filtered off. Red crystals of the title salt suitable for X-ray structure analysis were obtained from the filtered solution after a week.

### Refinement

The water H-atoms were located in a difference Fourier map, and were refined with distance restraints of O—H = 0.84 (1) and H···H = 1.37 (1) Å with *U*_iso_(H) = 1.5*U*_eq_(O). The other H-atoms were placed in calculated positions (C—H = 0.93–0.97 and O—H = 0.82 Å) and were included in the refinement in the riding model, with *U*_iso_(H) = 1.2*U*_eq_(aromatic C) and *U*_iso_(H) = 1.5*U*_eq_(hydroxyl O, methyl C).

### Crystal data

**Table:**

[Co(H_2_O)_6_](C_9_H_7_O_4_)_2_·2H_2_O	*F*(000) = 2344
*M**_r_* = 561.35	*D*_x_ = 1.546 Mg m^−^^3^
Orthorhombic, *I**b**c**a*	Mo *K*α radiation, λ = 0.71073 Å
Hall symbol: -I 2b 2c	Cell parameters from 7064 reflections
*a* = 10.6238 (10) Å	θ = 2.5–28.4°
*b* = 13.6271 (12) Å	µ = 0.79 mm^−^^1^
*c* = 33.318 (3) Å	*T* = 298 K
*V* = 4823.5 (8) Å^3^	Block, red
*Z* = 8	0.40 × 0.30 × 0.30 mm

### Data collection

**Table:**

Bruker APEXII CCD area-detector diffractometer	3036 independent reflections
Radiation source: fine-focus sealed tube	2508 reflections with *I* > 2σ(*I*)
graphite	*R*_int_ = 0.025
φ and ω scans	θ_max_ = 28.5°, θ_min_ = 2.5°
Absorption correction: multi-scan (*SADABS*; Bruker, 2008)	*h* = −14→10
*T*_min_ = 0.743, *T*_max_ = 0.798	*k* = −18→18
19647 measured reflections	*l* = −44→44

### Refinement

**Table:**

Refinement on *F*^2^	Primary atom site location: structure-invariant direct methods
Least-squares matrix: full	Secondary atom site location: difference Fourier map
*R*[*F*^2^ > 2σ(*F*^2^)] = 0.038	Hydrogen site location: inferred from neighbouring sites
*wR*(*F*^2^) = 0.110	H atoms treated by a mixture of independent and constrained refinement
*S* = 1.03	*w* = 1/[σ^2^(*F*_o_^2^) + (0.0555*P*)^2^ + 4.7025*P*] where *P* = (*F*_o_^2^ + 2*F*_c_^2^)/3
3036 reflections	(Δ/σ)_max_ = 0.001
184 parameters	Δρ_max_ = 0.50 e Å^−^^3^
12 restraints	Δρ_min_ = −0.38 e Å^−^^3^

### Special details

**Table:**

Geometry. All e.s.d.'s (except the e.s.d. in the dihedral angle between two l.s. planes) are estimated using the full covariance matrix. The cell e.s.d.'s are taken into account individually in the estimation of e.s.d.'s in distances, angles and torsion angles; correlations between e.s.d.'s in cell parameters are only used when they are defined by crystal symmetry. An approximate (isotropic) treatment of cell e.s.d.'s is used for estimating e.s.d.'s involving l.s. planes.
Refinement. Refinement of *F*^2^ against ALL reflections. The weighted *R*-factor *wR* and goodness of fit *S* are based on *F*^2^, conventional *R*-factors *R* are based on *F*, with *F* set to zero for negative *F*^2^. The threshold expression of *F*^2^ > σ(*F*^2^) is used only for calculating *R*-factors(gt) *etc*. and is not relevant to the choice of reflections for refinement. *R*-factors based on *F*^2^ are statistically about twice as large as those based on *F*, and *R*- factors based on ALL data will be even larger.

### Fractional atomic coordinates and isotropic or equivalent isotropic displacement parameters (Å^2^)

**Table:**

	*x*	*y*	*z*	*U*_iso_*/*U*_eq_	
Co1	0.5000	0.2500	0.466722 (9)	0.04068 (13)	
O1	0.51255 (17)	0.35551 (13)	0.51203 (5)	0.0568 (4)	
O2	0.69453 (14)	0.22453 (12)	0.46340 (4)	0.0470 (4)	
O3	0.52582 (14)	0.36122 (13)	0.42432 (4)	0.0517 (4)	
H1A	0.555 (2)	0.351 (2)	0.5330 (5)	0.078*	
H1B	0.4513 (18)	0.3927 (19)	0.5155 (7)	0.078*	
H2A	0.736 (2)	0.2629 (15)	0.4486 (7)	0.078*	
H2B	0.733 (2)	0.1720 (12)	0.4664 (8)	0.078*	
H3A	0.5802 (17)	0.358 (2)	0.4063 (6)	0.078*	
H3B	0.4574 (13)	0.380 (2)	0.4151 (7)	0.078*	
O4	0.84476 (13)	0.33748 (13)	0.41891 (4)	0.0533 (4)	
O5	0.70791 (12)	0.34819 (12)	0.36892 (4)	0.0487 (4)	
O6	1.29926 (13)	0.40867 (11)	0.38932 (4)	0.0477 (3)	
O7	0.77990 (15)	0.36935 (14)	0.29743 (4)	0.0608 (4)	
H7	0.7300	0.3635	0.3161	0.091*	
C1	0.81924 (17)	0.35113 (14)	0.38283 (5)	0.0376 (4)	
C2	0.92337 (16)	0.36890 (12)	0.35340 (5)	0.0333 (3)	
C3	1.04694 (17)	0.37693 (13)	0.36600 (5)	0.0335 (3)	
H3	1.0643	0.3756	0.3934	0.040*	
C4	1.14660 (18)	0.38701 (13)	0.33896 (5)	0.0362 (4)	
C5	1.1187 (2)	0.38921 (17)	0.29793 (6)	0.0482 (5)	
H5	1.1836	0.3943	0.2793	0.058*	
C6	0.9973 (2)	0.3840 (2)	0.28495 (5)	0.0557 (6)	
H6	0.9804	0.3872	0.2576	0.067*	
C7	0.89775 (19)	0.37410 (15)	0.31202 (5)	0.0424 (4)	
C8	1.27713 (17)	0.39131 (13)	0.35418 (6)	0.0387 (4)	
C9	1.3838 (2)	0.37177 (18)	0.32548 (7)	0.0552 (5)	
H9A	1.3879	0.4236	0.3060	0.083*	
H9B	1.3700	0.3103	0.3121	0.083*	
H9C	1.4617	0.3690	0.3401	0.083*	
O8	0.3217 (2)	0.47763 (16)	0.53449 (6)	0.0864 (7)	
H8A	0.290 (4)	0.465 (2)	0.5567 (6)	0.130*	
H8B	0.351 (4)	0.5344 (15)	0.5342 (10)	0.130*	

### Atomic displacement parameters (Å^2^)

**Table:**

	*U*^11^	*U*^22^	*U*^33^	*U*^12^	*U*^13^	*U*^23^
Co1	0.0320 (2)	0.0640 (3)	0.02606 (18)	−0.01256 (16)	0.000	0.000
O1	0.0641 (11)	0.0646 (10)	0.0417 (8)	0.0001 (8)	−0.0173 (7)	−0.0097 (7)
O2	0.0382 (7)	0.0689 (10)	0.0339 (7)	−0.0131 (7)	−0.0052 (5)	0.0096 (6)
O3	0.0347 (7)	0.0834 (11)	0.0371 (7)	−0.0033 (7)	0.0018 (5)	0.0135 (7)
O4	0.0369 (7)	0.0905 (11)	0.0325 (7)	−0.0114 (7)	0.0012 (5)	0.0148 (7)
O5	0.0309 (7)	0.0726 (10)	0.0424 (7)	−0.0018 (6)	−0.0021 (5)	0.0050 (6)
O6	0.0362 (7)	0.0680 (9)	0.0390 (7)	0.0004 (6)	0.0033 (5)	0.0039 (6)
O7	0.0472 (9)	0.0981 (13)	0.0372 (7)	0.0024 (8)	−0.0107 (6)	0.0048 (8)
C1	0.0331 (9)	0.0456 (10)	0.0341 (8)	−0.0020 (7)	0.0016 (7)	0.0042 (7)
C2	0.0353 (9)	0.0362 (8)	0.0286 (8)	0.0016 (7)	0.0010 (7)	0.0026 (6)
C3	0.0361 (9)	0.0383 (8)	0.0261 (7)	0.0004 (7)	0.0029 (6)	0.0028 (6)
C4	0.0377 (9)	0.0390 (9)	0.0319 (8)	0.0014 (7)	0.0059 (7)	0.0038 (7)
C5	0.0494 (12)	0.0654 (13)	0.0299 (8)	0.0022 (10)	0.0098 (8)	0.0049 (8)
C6	0.0606 (14)	0.0819 (16)	0.0247 (8)	0.0037 (11)	−0.0001 (8)	0.0047 (9)
C7	0.0430 (10)	0.0520 (10)	0.0321 (9)	0.0043 (8)	−0.0046 (7)	0.0031 (8)
C8	0.0372 (10)	0.0397 (9)	0.0391 (9)	−0.0002 (7)	0.0101 (7)	0.0073 (7)
C9	0.0421 (11)	0.0695 (14)	0.0542 (12)	−0.0008 (10)	0.0184 (9)	−0.0014 (10)
O8	0.0901 (16)	0.0714 (13)	0.0976 (16)	0.0081 (11)	0.0233 (12)	0.0315 (11)

### Geometric parameters (Å, °)

**Table:**

Co1—O1	2.0888 (15)	C1—C2	1.498 (2)
Co1—O1^i^	2.0888 (15)	C2—C3	1.383 (3)
Co1—O2	2.0985 (16)	C2—C7	1.407 (2)
Co1—O2^i^	2.0985 (16)	C3—C4	1.397 (2)
Co1—O3^i^	2.0900 (15)	C3—H3	0.9300
Co1—O3	2.0900 (15)	C4—C5	1.399 (3)
O1—H1A	0.84 (1)	C4—C8	1.478 (3)
O1—H1B	0.83 (1)	C5—C6	1.362 (3)
O2—H2A	0.84 (1)	C5—H5	0.9300
O2—H2B	0.83 (1)	C6—C7	1.396 (3)
O3—H3A	0.84 (1)	C6—H6	0.9300
O3—H3B	0.83 (1)	C8—C9	1.507 (3)
O4—C1	1.246 (2)	C9—H9A	0.9600
O5—C1	1.271 (2)	C9—H9B	0.9600
O6—C8	1.217 (2)	C9—H9C	0.9600
O7—C7	1.345 (2)	O8—H8A	0.83 (1)
O7—H7	0.8200	O8—H8B	0.835 (10)
			
O1—Co1—O1^i^	87.46 (10)	C3—C2—C7	118.51 (16)
O1—Co1—O3^i^	174.85 (6)	C3—C2—C1	121.00 (15)
O1^i^—Co1—O3^i^	88.91 (7)	C7—C2—C1	120.45 (16)
O1—Co1—O3	88.91 (7)	C2—C3—C4	122.11 (16)
O1^i^—Co1—O3	174.85 (6)	C2—C3—H3	118.9
O3^i^—Co1—O3	94.94 (9)	C4—C3—H3	118.9
O1—Co1—O2	95.11 (6)	C3—C4—C5	118.12 (18)
O1^i^—Co1—O2	89.26 (6)	C3—C4—C8	119.59 (15)
O3^i^—Co1—O2	88.49 (6)	C5—C4—C8	122.24 (16)
O3—Co1—O2	87.42 (6)	C6—C5—C4	120.67 (18)
O1—Co1—O2^i^	89.26 (6)	C6—C5—H5	119.7
O1^i^—Co1—O2^i^	95.11 (6)	C4—C5—H5	119.7
O3^i^—Co1—O2^i^	87.42 (6)	C5—C6—C7	121.13 (17)
O3—Co1—O2^i^	88.49 (6)	C5—C6—H6	119.4
O2—Co1—O2^i^	173.95 (7)	C7—C6—H6	119.4
Co1—O1—H1A	125.8 (18)	O7—C7—C6	118.45 (17)
Co1—O1—H1B	118.0 (18)	O7—C7—C2	122.13 (18)
H1A—O1—H1B	110.7 (16)	C6—C7—C2	119.42 (18)
Co1—O2—H2A	116.2 (18)	O6—C8—C4	121.26 (16)
Co1—O2—H2B	129 (2)	O6—C8—C9	119.95 (18)
H2A—O2—H2B	110.4 (16)	C4—C8—C9	118.77 (17)
Co1—O3—H3A	122.3 (19)	C8—C9—H9A	109.5
Co1—O3—H3B	111.3 (19)	C8—C9—H9B	109.5
H3A—O3—H3B	110.9 (16)	H9A—C9—H9B	109.5
C7—O7—H7	109.5	C8—C9—H9C	109.5
O4—C1—O5	123.33 (17)	H9A—C9—H9C	109.5
O4—C1—C2	119.66 (16)	H9B—C9—H9C	109.5
O5—C1—C2	116.97 (15)	H8A—O8—H8B	111.0 (17)
			
O4—C1—C2—C3	−4.4 (3)	C5—C6—C7—O7	−180.0 (2)
O5—C1—C2—C3	177.55 (17)	C5—C6—C7—C2	−0.3 (4)
O4—C1—C2—C7	173.23 (18)	C3—C2—C7—O7	−178.33 (18)
O5—C1—C2—C7	−4.9 (3)	C1—C2—C7—O7	4.0 (3)
C7—C2—C3—C4	−1.9 (3)	C3—C2—C7—C6	2.0 (3)
C1—C2—C3—C4	175.68 (17)	C1—C2—C7—C6	−175.7 (2)
C2—C3—C4—C5	0.2 (3)	C3—C4—C8—O6	−16.9 (3)
C2—C3—C4—C8	−177.58 (16)	C5—C4—C8—O6	165.37 (19)
C3—C4—C5—C6	1.6 (3)	C3—C4—C8—C9	161.89 (18)
C8—C4—C5—C6	179.3 (2)	C5—C4—C8—C9	−15.8 (3)
C4—C5—C6—C7	−1.5 (4)		

Symmetry codes: (i) −*x*+1, −*y*+1/2, *z*.

### Hydrogen-bond geometry (Å, °)

**Table:**

*D*—H···*A*	*D*—H	H···*A*	*D*···*A*	*D*—H···*A*
O1—H1A···O4^ii^	0.84 (1)	1.93 (1)	2.766 (2)	179 (3)
O1—H1B···O8	0.83 (1)	1.91 (1)	2.728 (3)	168 (2)
O2—H2A···O4	0.84 (1)	1.83 (1)	2.667 (2)	172 (3)
O2—H2B···O8^iii^	0.83 (1)	2.25 (1)	3.069 (3)	171 (3)
O3—H3A···O5	0.84 (1)	1.85 (1)	2.680 (2)	176 (2)
O3—H3B···O6^iv^	0.83 (1)	1.93 (1)	2.752 (2)	172 (3)
O7—H7···O5	0.82	1.79	2.518 (2)	147.
O8—H8A···O6^ii^	0.83 (1)	2.17 (1)	2.996 (2)	171 (3)
O8—H8B···O1^v^	0.84 (1)	2.59 (2)	3.267 (3)	139 (3)
O8—H8B···O3^v^	0.84 (1)	2.37 (3)	3.054 (3)	139 (3)

Symmetry codes: (ii) −*x*+3/2, *y*, −*z*+1; (iii) *x*+1/2, −*y*+1/2, −*z*+1; (iv) *x*−1, *y*, *z*; (v) −*x*+1, −*y*+1, −*z*+1.
